# Notes and News

**Published:** 1901-02-02

**Authors:** 


### NOTES AND NEWS.

OWING to the death of Queen Victoria, the dinner arranged by the Royal College of Surgeons of England in connection with the Hunterian Festival, for February 14th, will not take place. The Hunterian oration will be delivered by Mr. N. C. Macnamara at 4 o'clock p.m. on that day.

MR. W. OXENDEN HAMMOND has made another donation of £500 to the Middlesex Hospital towards the investigation of cancer.

THE appearance of typhoid fever in San Remo has been traced to a particular source of milk supply. Careful inspection and supervision have been exercised since, and no further cases are reported.

A CRY, which has almost attained political importance, has been raised for "pure beer," and what is meant by this term is beer brewed from malt and hops alone. It may be well then to remember, as is pointed out by the *Polyclinic*, that hops were at one time a forbidden adulteration.

A BEQUEST has been made by the late Rev. J. Gerard Young to secure the establishment and maintenance of a cottage hospital for Monifieth in Scotland. A proposal to secure a cottage hospital for four beds, on a site rented from the Earl of Dalhousie, is before the Court of Session at Dundee. The bequest amounts to £7,850.

MR. BURTON, formerly chemist of the well-known firm of Wedgwood, Manchester, speaking at the annual dinner of the English China Manufacturers' Association, took up the subject of lead poisoning in the china trade. He said that English manufacturers were prepared to take every precaution to prevent this occurring amongst employés, but that the adoption of any leadless glaze now in use was practically impossible. If the English public bought foreign products in preference to British made, under the impression that more care was exercised upon the Continent, they were making a great mistake, as it was not so. We think, however, there is another side to the question. Very strong evidence has been given lately as to the practicability of leadless glazes.

A PASTEUR Department has been established at the Baltimore City Hospital by the College of Physicians and Surgeons of that city.

A HYDROPATHIC Institute has been attached to the Berlin University, it is installed in one of the new Charité pavilions, and Professor Brieger has undertaken the direction.

THE late Lady Freake has bequeathed £100 to St. George's Hospital, St. John's Hospital, Twickenham, and the Victoria Hospital for Children, Chelsea, and the late Mrs. May Coles has left £3,000 between the Gravesend Hospital and St. Bartholomew's Hospital, Rochester.

ANOTHER death from the plague has occurred at Hull, making the seventh victim from the "Friary." Hull has been declared an infected port by the Netherlands Government. "The 'Friary' is now out of quarantine." A vessel has arrived at Bristol upon which some rats were discovered to have died of plague, and the ship and its crew have consequently been placed under observation.

THE new buildings of the Northern Hospital in Liverpool will probably be opened in the spring in a formal manner. The building is now in occupation, but one ward remains untenanted owing to lack of funds. The hospital is to bear the name of the David Lewis Northern Hospital, to identify it with the benefactor through whose bequest it was possible to erect a new institution.

THERE are not many localities nowadays which neglect to provide accommodation for infectious cases, and, moreover, most local authorities are now sufficiently wise to avail themselves of the advantage of a joint institution serving several districts. But there are yet a few laggards in the field, and their sins of omission may any day be brought home to them. This is the case with Burghead, in Morayshire, where some cases of smallpox have appeared. Burghead refused to join in the provision of an infectious diseases hospital for the burgh of Elgin. Now they are compelled to ask for accommodation in this hospital, and they must pay five guineas a week for each patient. If the outbreak spreads no further the expense will not after all be very considerable, but if it does, Burghead may learn now that amalgamation comes cheapest in the end.

ON January 14th a meeting was held at Oldham to discuss the question of extending the Infirmary, a project which was chosen in 1897 to commemorate the Diamond Jubilee. The original scheme required an estimated expenditure of £16,000, and as the collection fell considerably short of this sum the work has not been commenced. It was remarked at the meeting that if the scheme were not soon put in hand the commemoration would be celebrated after the demise of the Queen. It was also pointed out that Oldham should be expected to raise a sum worthy of its importance, and that the very necessary work of improving the Infirmary might be commenced with confidence with £11,000 in hand, relying on the activity of the efforts being made to collect the rest of the necessary funds. At the meeting it was decided to proceed with the scheme. Those who are taking part in the project have now an added call to perpetuate the memory of Queen Victoria.

